# Electric Field Characteristics of Rotating Permanent Magnet Stimulation

**DOI:** 10.3390/bioengineering11030258

**Published:** 2024-03-06

**Authors:** Pei L. Robins, Sergey N. Makaroff, Michael Dib, Sarah H. Lisanby, Zhi-De Deng

**Affiliations:** 1 Computational Neurostimulation Research Program, Noninvasive Neuromodulation Unit, Experimental Therapeutics and Pathophysiology Branch, National Institute of Mental Health, Bethesda, MD 20892, USA; pei.robins@nih.gov (P.L.R.); sarah.lisanby@nih.gov (S.H.L.); 2 Department of Electrical and Computer Engineering, Worcester Polytechnic Institute, Worcester, MA 01609, USA; snmakaroff@wpi.edu; 3 Fischell Department of Bioengineering, University of Maryland, College Park, MD 20742, USA; msdib98@gmail.com

**Keywords:** electric field, finite element method, permanent magnets, head phantom measurement, rotating magnets, magnetic stimulation, neuromodulation, depression

## Abstract

Neurostimulation devices that use rotating permanent magnets are being explored for their potential therapeutic benefits in patients with psychiatric and neurological disorders. This study aims to characterize the electric field (E-field) for ten configurations of rotating magnets using finite element analysis and phantom measurements. Various configurations were modeled, including single or multiple magnets, and bipolar or multipolar magnets, rotated at 10, 13.3, and 350 revolutions per second (rps). E-field strengths were also measured using a hollow sphere (r=9.2 cm) filled with a 0.9% sodium chloride solution and with a dipole probe. The E-field spatial distribution is determined by the magnets’ dimensions, number of poles, direction of the magnetization, and axis of rotation, while the E-field strength is determined by the magnets’ rotational frequency and magnetic field strength. The induced E-field strength on the surface of the head ranged between 0.0092 and 0.52 V/m. In the range of rotational frequencies applied, the induced E-field strengths were approximately an order or two of magnitude lower than those delivered by conventional transcranial magnetic stimulation. The impact of rotational frequency on E-field strength represents a confound in clinical trials that seek to tailor rotational frequency to individual neural oscillations. This factor could explain some of the variability observed in clinical trial outcomes.

## 1. Introduction

Conventional magnetic stimulation systems, such as transcranial magnetic stimulation (TMS), utilize a current-carrying coil to generate a time-varying magnetic field pulse. This process produces a spatially varying electric field (E-field)—via electromagnetic induction—in the central or peripheral nervous system. TMS is cleared by the United States Food and Drug Administration (FDA) for major depression, anxious depression, obsessive–compulsive disorder, smoking cessation, and migraines [1,2]. An alternative approach to generating a time-varying magnetic field involves mechanically rotating permanent magnets. Several rotating magnet devices have been proposed [3,4,5], using rotating high-strength neodymium magnets to induce an E-field in nearby nerve tissue. The strength, efficiency, and precision of these rotating magnets in inducing E-fields for use in neuromodulation have yet to be established.

**Figure 1:**
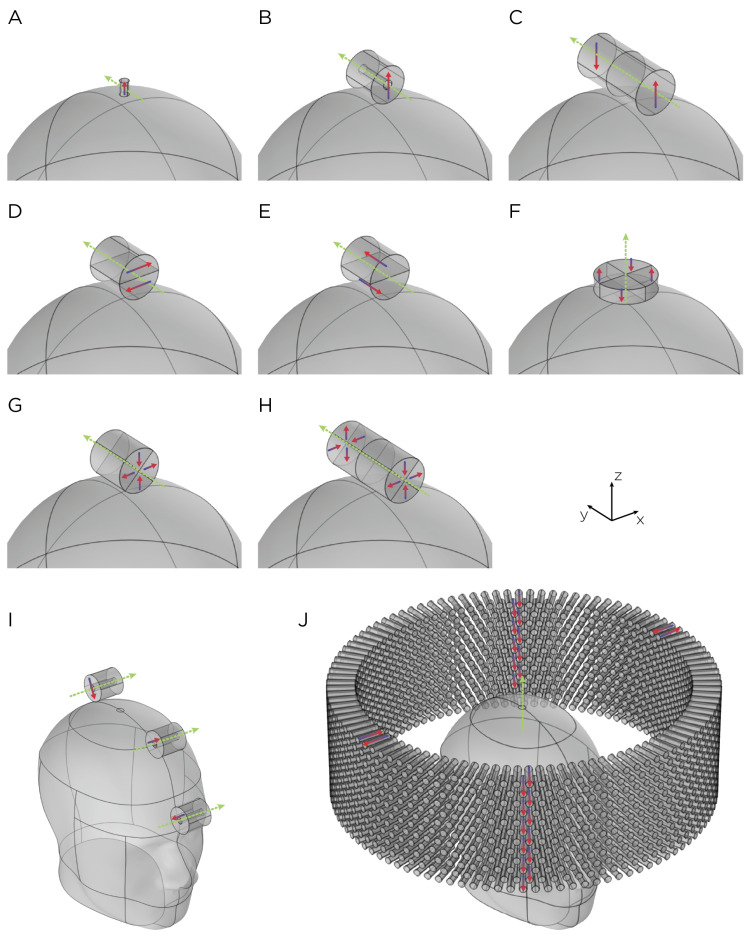
Dimensions, placement, and magnetization directions for ten configurations of rotating magnets (**A–J**). Model of single magnets in the (**A**) TRPMS and (**B**) sTMS systems. (**C**–**H**) Model of single magnets with multiple segments of different magnetization directions. (**I**) Model of the full sTMS system. (**J**) Model of the wide-bore, low-frequency magnetic spinner. The green arrows show the rotation axes, with the rotation direction determined by the right-hand rule. The red/blue arrows show the direction of the magnetization.

One such system, known as synchronized transcranial magnetic stimulation (sTMS) or Neuro-EEG Synchronization Therapy (NEST), has been investigated as an innovative approach to personalize the treatment of major depressive disorder (MDD) [6,7,8,9,10]. The sTMS device consists of three cylindrical N52 grade neodymium magnets (Figure 1; Model I), which are diametrically magnetized with a surface field of 0.64 T [3,7,9,11]. The magnets rotate along the cylindrical axis and are positioned over the midline frontal polar brain region, the superior frontal gyrus, and the parietal region. The rotation speed of the magnets is customized to match the patient’s individual alpha frequency (IAF) of neural oscillations, as determined by pre-treatment electroencephalography (EEG) recorded from a fronto-occipital montage while the patient is in an eyes-closed resting state [9]. The hypothesized mechanism of action involves the entrainment of alpha oscillations through exogenous subthreshold sinusoidal stimulation produced by sTMS. This aims to reset the neural oscillators, enhance cortical plasticity, normalize cerebral blood flow, and thereby ameliorate depressive symptoms [6]. In contrast to conventional TMS, the sTMS device delivers a sinusoidal and subthreshold intensity stimulus.

In a multicenter, double-blinded, sham-controlled clinical trial evaluating the efficacy of sTMS for the treatment of depression, no significant difference was observed between the active and sham in the intent-to-treat (ITT) analysis [7]. However, among patients who completed the treatment per-protocol, there was a significant treatment response after six weeks. The authors also showed that patients in the per-protocol treatment group with a history of poor response or failed medication trials had a better improvement compared to those who received no prior treatment, suggesting that more severely depressed patients may benefit more from sTMS treatment. Additionally, secondary analysis showed that a lower IAF correlated with a lower treatment response [8]. In addition to MDD, sTMS has also been explored as a therapeutic intervention for post-traumatic stress disorder (PTSD) [12]. In a small prospective, sham-controlled, multisite pilot of sTMS treatment for patients experiencing moderate-to-severe symptoms of PTSD, there was a greater reduction in the PTSD threshold symptoms [12]. However, there was no significant difference between the active and sham groups. Furthermore, ongoing research is assessing the safety and feasibility of sTMS in individuals with cocaine, opioid, and alcohol use disorders (ClinicalTrials.gov Identifier: NCT04336293).

Another device that employs similar mechanics is the transcranial rotating permanent magnet stimulator (TRPMS) [5,13,14]. This portable, battery-operated device consists of an array of small cylindrical N52-grade neodymium magnets mounted on high-speed motors, which are in turn mounted on a helmet. Compared to the sTMS device, the TRPMS device uses smaller magnets, measuring 0.9525 cm in height and 0.635 cm in diameter, but has a stronger remanent magnetic flux density ($B_{r}=1.48\;T$). In addition, the TRPMS magnets are axially magnetized, whereas the sTMS magnets are diametrically magnetized. However, the axis of rotation for the TRPMS magnets is perpendicular to the cylindrical axis of the magnet, whereas in the sTMS system, the axis of rotation is parallel to the cylindrical axis of the magnet. The motor operates at a no-load speed of 24,000 revolutions per minute (rpm) or 400 revolutions per second (rps), achieving a rotational speed of 20,000 rpm (approximately 333 rps) under load. The induced E-field strength is directly proportional to the rotational frequency of the magnet, a higher rotational speed of the TRPMS magnets results in a higher E-field strength compared to the sTMS system. Voltage measurements conducted by Helekar and colleagues used an inductor search coil to estimate the maximum intensity of the TRPMS device to be approximately 7% of that produced by the maximum conventional TMS output [14]. At a distance of 21.2 to 26.2 mm from the TRPMS and inductor, representing the depth of the cerebral cortex, the intensity reduces by approximately half.

Recent studies have shown the safety and potential effectiveness of the TRPMS device in treating voiding dysfunction in patients with multiple sclerosis (MS) [15,16,17,18]. In a feasibility and safety study, the microstimulators from the TRPMS device were individually placed over predetermined regions of interest (ROI) during voiding initiation [15,17,18]. These predetermined ROIs were identified from the individual blood-oxygen-level-dependent (BOLD) activation at voiding initiation. Applying the TRPMS device to brain regions that modulate voiding initiation significantly improved bladder emptying symptoms [15,17,18]. Additionally, a proof-of-concept pilot study suggests that the TRPMS device may offer potential benefits for muscle function in individuals with type 1 myotonic dystrophy [16].

Yet another system that uses a magnet array is a wide-bore, low-frequency magnetic spinner comprising approximately 1300 Alnico permanent magnets [19]. These magnets are arranged radially within a 30 cm diameter ring (Figure 1; Model J). The resulting rotating magnetic field is perpendicular to the ring axis, in which the measured magnetic field strength at the center of the bore is approximately 32 mT. The device reaches a rotational speed up to 15 rps. This wide-bore magnetic spinner was originally designed to induce alternating electric currents in biological tissues, particularly in bones. Its application for brain stimulation has yet to be evaluated.

The utilization of rotating magnets has also been proposed for the stimulation of peripheral nerves and muscles [20]. Recognizing that long, straight nerves are more responsive to E-field gradients, Watterson proposed the use of multipole magnets with different magnetization directions and different axes of rotation to achieve a higher field gradient [4,20]. In a series of in vitro experiments, Watterson employed a bipole configuration, featuring two diametrically magnetized cylindrical segments (N52 grade neodymium magnets with a surface field ranging from 1.43 T to 1.48 T), positioned adjacent to one another with opposite magnetization directions, to activate the cane toad sciatic nerve and the attached gastrocnemius muscle [20]. It was demonstrated that muscle and nerve activation could be achieved with rotational frequencies of 180 rps and 230 rps, respectively.

In this work, we assess the E-field characteristics of various rotating magnet configurations through computational modeling. Complementary to numerical simulations, experimental measurements of field strengths are performed on a head phantom, validating the computational results. Our objective is to provide a comprehensive and comparative understanding of the E-field profiles generated by different rotating magnet setups. We further compare their E-field characteristics to those generated by conventional TMS. Via a combination of computational simulations and experimental validation, this comparative analysis aims to elucidate a comprehensive understanding of the potential advantages and limitations offered by rotating magnets for noninvasive brain stimulation applications.

## 2. Methods

### 2.1. Simulations and Solver

The finite element models were implemented in COMSOL Multiphysics (COMSOL, Burlington, MA, USA). Two different head models were used: a spherical head with a radius of 8.5 cm (Model A–H) and the Institute of Electrical and Electronics Engineers’ (IEEEs) Specific Anthropomorphic Mannequin (SAM) phantom head (Model I–J), as illustrated in Figure 1. Both the sphere and SAM phantom head were characterized by uniform, isotropic electrical conductivity, σ=0.33 S/m, and relative permeability, $\mu_{r}=1$. In a homogeneous, symmetric conductor head model, the E-field induced by magnetic stimulation is tangential to the surface of the head model. The E-field is insensitive to radial variations of conductivity. This has been shown mathematically for low frequencies that are generally used for transcranial stimulation of the brain [21]. Therefore, the exact conductivity value used in our head model is not expected to affect the E-field. The tissue relative permittivity at low frequencies is approximately $1\times 10^{7}$ [22,23], although this parameter does not affect our quantity of interest. The magnets are cylindrical; they have recoil permeability, $\mu_{\mathrm{rec}}=1.05$, which is typical of neodymium magnets [24]. The recoil permeability is the slope of the linear portion of the *B*-*H* curve, where *B* is the magnetic flux density and *H* is the magnetic field strength (see neodymium magnet demagnetization curves in [24]). The rotor—the moving components of the system—includes the magnet(s); the stator—the stationary part of the system—includes the head model and the surrounding air sphere.

Under the magnetic vector potential (A–*V*) formulation and the induced solenoidal E-field, Ampère’s law was applied to all domains:

(1) $$\sigma \frac{\partial A}{\partial t}+\nabla \times \left(\frac{1}{\mu }\nabla \times A\right)=0.$$

This equation signifies the relationship between the time-varying component of the magnetic vector potential (A), the material’s conductivity (σ), and its permeability (μ). Additionally, for the sections of both the rotor and stator that were devoid of current, a magnetic flux conservation equation pertinent to the scalar magnetic potential was applied. This equation is represented as:

(2) $$-\nabla \;\cdot \;\left(\mu \nabla V_{m}-B_{r}\right)=0.$$

Here, $V_{m}$ denotes the magnetic scalar potential, while $B_{r}$ represents the remanent magnetic flux density, as detailed in [25]. Furthermore, to maintain consistency, the continuity of the scalar magnetic potential was ensured at the interface between the rotor and stator.

The stator and rotor were meshed, and then the stationary solution was obtained using the multifrontal massively parallel sparse direct solver (MUMPS). The time-dependent problem was then solved in 10° rotation steps, using a relative tolerance of $1.0\times 10^{-8}$. This approach is based on the assumption that the transient effects originating from the initiation of the rotating magnets have diminished. Consequently, the obtained final solution is indicative of the system’s steady-state behavior.

### 2.2. Magnet Configuration

The magnets in each model are cylindrically shaped (Figure 1). Models A and B represent single magnets from the TRPMS and sTMS systems, respectively. Model A, which measures 0.9525 cm in height and 0.635 cm in diameter, has an axial magnetization and a residual flux density of 1.48 T. This magnet is rotated around its diameter axis and tangentially to the spherical head at 350 rps. Model B measures 2.54 cm in height and diameter, with an inner diameter of 0.635 cm. The magnet is diametrically magnetized with a residual flux density of 1.32 T and rotates about its central axis at 10 rps. To confirm that the E-field strength is linearly proportional to the rotational frequency of the magnet, we performed a parametric simulation using Model A, varying the rotational frequency from 10 rps to 400 rps.

Models C–H represent multipole configurations [4]. Model C is a bipolar magnet configuration, consisting of two diametrically magnetized cylindrical segments, each segment measures 3 cm in height and diameter, placed adjacent to each other with opposite magnetization. Model D is another bipolar configuration (3 cm in height and diameter), consisting of two diametrically magnetized, half-cylindrical segments with opposite magnetization directions. Model E (3 cm in height and diameter), similar to Model D, consists of two axially magnetized, half-cylindrical segments with opposite magnetization directions. Model F is a quadrupolar configuration (1 cm in height and 5 cm in diameter), consisting of four quadrants axially magnetized with each quadrant alternating and opposite magnetization around the central axis. The configuration is positioned on the base of the cylindrical configuration and rotates around its central axis. Model G is a quadrupolar configuration (3 cm in height and diameter), consisting of four quadrants radially magnetized with each quadrant alternating and opposite magnetization around the central axis. Model H’s configuration utilizes eight segments (6 cm in height and 3 cm in diameter), in which two Model G-like configurations are placed adjacent to each other, ensuring all eight quadrants have opposite magnetization. Configuration C–H has a residual flux density of 1.48 T and rotates around its central axis at 10 rps.

Model I depicts the complete sTMS system, which includes three cylindrical magnets aligned along the sagittal midline of the head. The positioning of these magnets is as follows: The frontmost magnet is situated above the frontal pole, above the eyebrows; the middle magnet, positioned 7.1 cm from the frontmost magnet, aligns approximately with the superior frontal gyrus; and the most posterior magnet, located 9.2 cm from the middle magnet, corresponds roughly to the parietal cortex area. Each magnet measures 2.54 cm in both diameter and height, with an inner diameter of 0.635 cm. They are diametrically magnetized and possess a residual flux density of 1.32 T. The rotation axes are oriented perpendicular to the sagittal plane, and the rotational frequency is 10 rps, mirroring the center frequency of the alpha band oscillation. Model J, on the other hand, represents a wide-bore, low-frequency magnetic spinner. This spinner is composed of 1224 cylindrical magnets, each 2.54 cm tall and 0.3175 cm in diameter. These magnets are axially magnetized and arranged radially within a ring with a 30 cm diameter. The magnets are uniformly distributed across 12 layers in a staggered stacking formation, with each layer being 1.905 cm apart. The spinner operates at a rotational frequency of 13.3 rps.

### 2.3. E-Field Measurements

**Figure 2:**
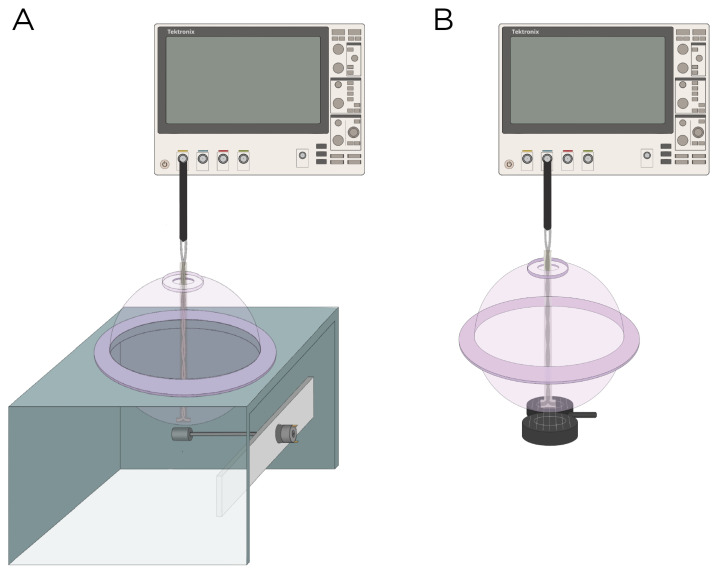
Experimental setup to measure the induced E-field strength using (**A**) rotating magnets Models A and B and (**B**) the MagVenture TMS coil.

The E-field was characterized experimentally using a hollow sphere mold with a radius of 9.2 cm (Ibili, Bergara, Spain) as the head phantom, along with a custom-made silver–chloride (AgCl) twisted pair dipole probe [26]. The probe was constructed from 99.99% pure silver, 21 gauge wire, with a bare diameter of 0.635 mm, and coated with a 0.762 mm perfluoroalkoxy (PFA) layer. For insulation, the probe was coated in epoxy resin with a thickness of approximately 0.2 cm. The tips of probes are separated by a distance of 9.40 mm. The exposed tips of the probe were immersed in Clorox bleach until a light gray color was observed. The two hemispheres of the sphere mold were sealed with vacuum grease and were filled with approximately 3 L of 0.9% sodium chloride (NaCl) in deionized water to emulate the conductivity of the brain (3.33 mS/cm at 20 °C) [27]; previous research has shown that 0.9% NaCl has a conductivity of 12 mS/cm at 20 °C [28]. Figure 2 illustrates the measurement apparatus.

Model A and B were experimentally measured using magnets from K & J Magnets Inc (Pipersville, USA). (Figure 2A). The magnet in Model A was mounted perpendicular inside a cylinder-shaped polyetheretherketone (PEEK) material and attached to a 24 V motor (model RS550, Shengle Electronic, Quanzhou, China), enabling the magnet to rotate around its central axis and tangentially to the spherical head. The magnet in Model B had an aluminum rod attached to its inner diameter and positioned approximately 5.08 cm away from the motor to minimize interference between the magnet and the motor. Rotation of the magnet occurred along the axial direction of the cylinder. The revolution (period=T) of the magnets was measured using a digital hand tachometer (PH-200LC, Mitutoyo, Kawasaki, Japan) and a piece of reflective tape (0.64 cm×1.27 cm). In addition to the rotating magnets, the E-field was measured with the MagVenture TMS coil (figure-8, cooled B65 coil). The probe was oriented to measure the maximum E-field at 100% maximum output of a MagPro X100 stimulator (MagVenture A/S, Farum, Denmark) (Figure 2B).

## 3. Results

### 3.1. Simulations

**Figure 3:**
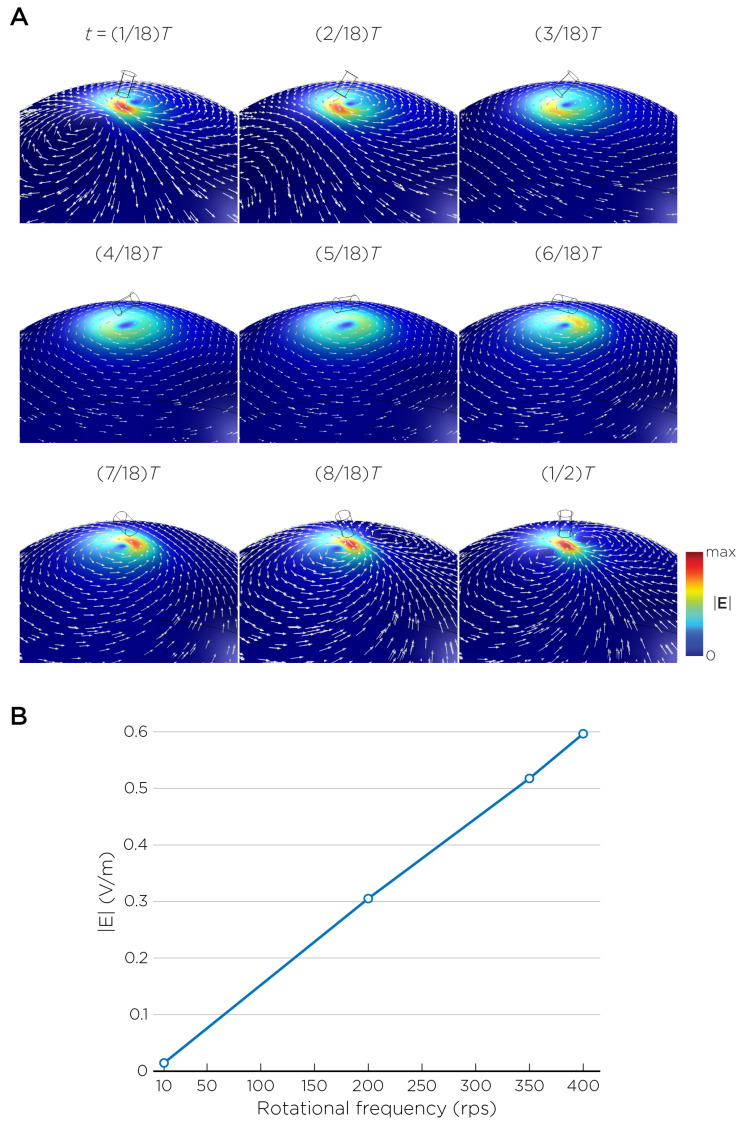
(**A**) Half revolution of configuration A in steady state. The cylinder represents the magnet. (**B**) The induced E-field strengths as a function of rotational frequencies.

**Figure 4:**
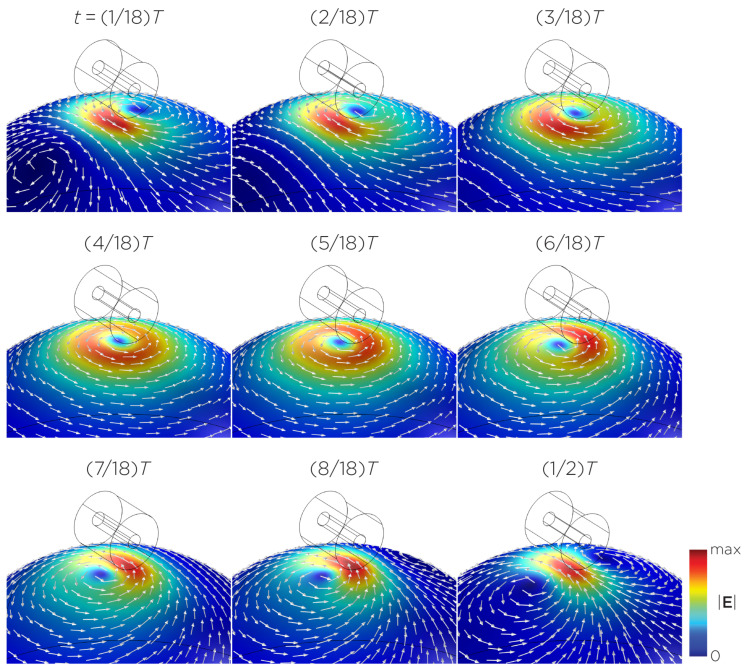
Half revolution of configuration B in steady state. The cylinder represents the magnet.

The computational parameters and the maximum induced E-field strength for Modela A–J are found in Table 1. Figure 3A illustrates the E-field distribution for Model A, representing the single rotating magnet in the TRPMS system. As the magnet rotates, the E-field distribution transitions from a figure-8 pattern (when the magnetic dipole is perpendicular to the spherical head at multiples of T/2) to a circular pattern (when the magnetic dipole aligns parallel to the head at multiples of T/4). The peak induced E-field strength at the surface of the head is approximately 0.52 V/m, in the direction parallel to the rotation axis of the magnet. In addition, the induced E-field strengths are linearly proportional to the rotational frequencies in the range of 10 rps to 400 rps (Figure 3B). Figure 4, representing the single magnet in the sTMS system (Model B), presents a similar E-field distribution to Figure 3 at a lower E-field strength. The peak induced E-field strength at the head’s surface for this magnet configuration measures approximately 0.098 V/m in the direction perpendicular to the direction magnetization.

**Figure 5:**
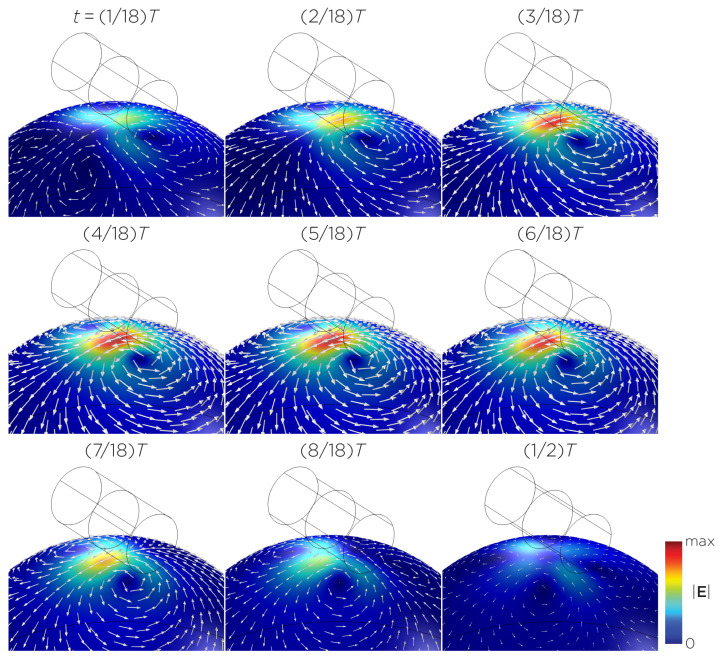
Half revolution of configuration C in steady state. The cylinder represents the magnet.

**Figure 6:**
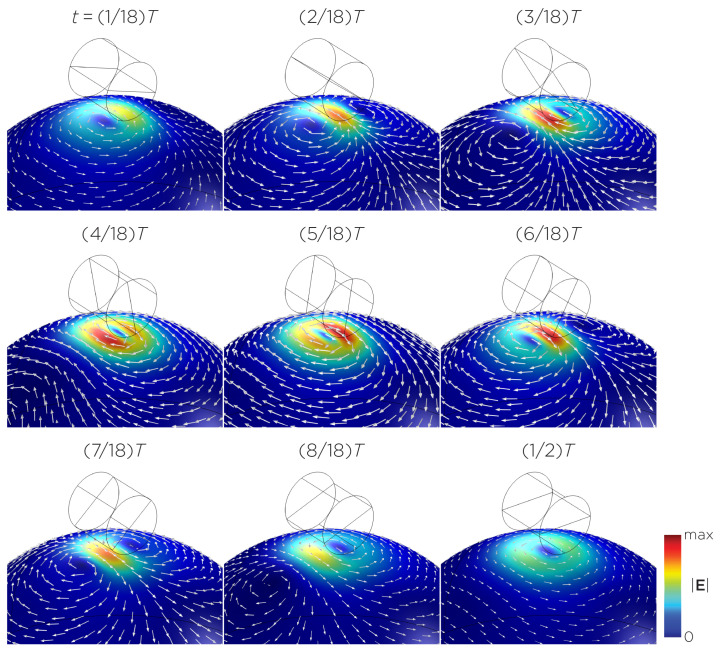
Half revolution of configuration D in steady state. The cylinder represents the magnet.

**Figure 7:**
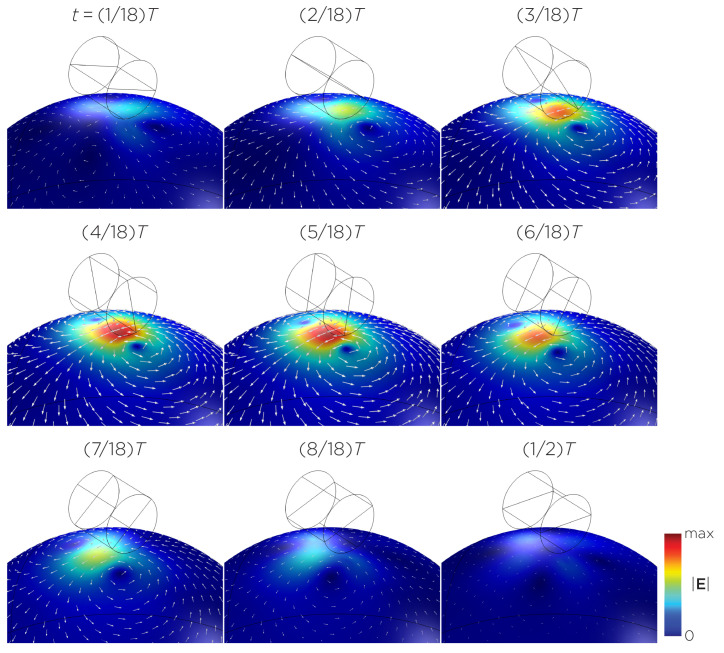
Half revolution of configuration E in steady state. The cylinder represents the magnet.

**Figure 8:**
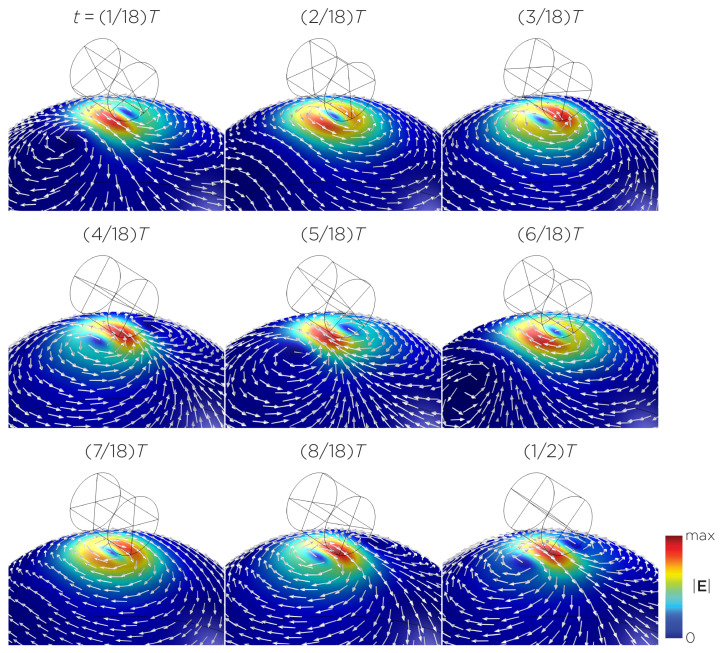
Half revolution of configuration F in steady state. The cylinder represents the magnet.

**Figure 9:**
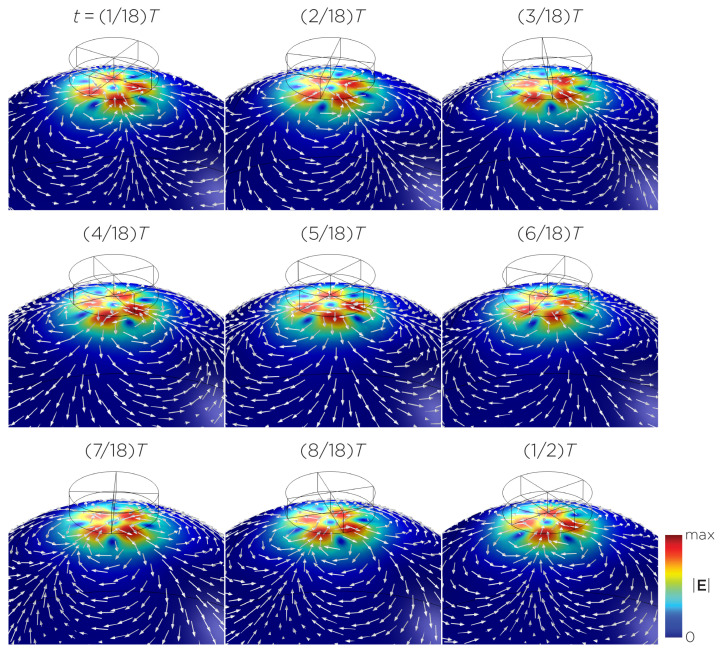
Half revolution of configuration G in steady state. The cylinder represents the magnet.

**Figure 10:**
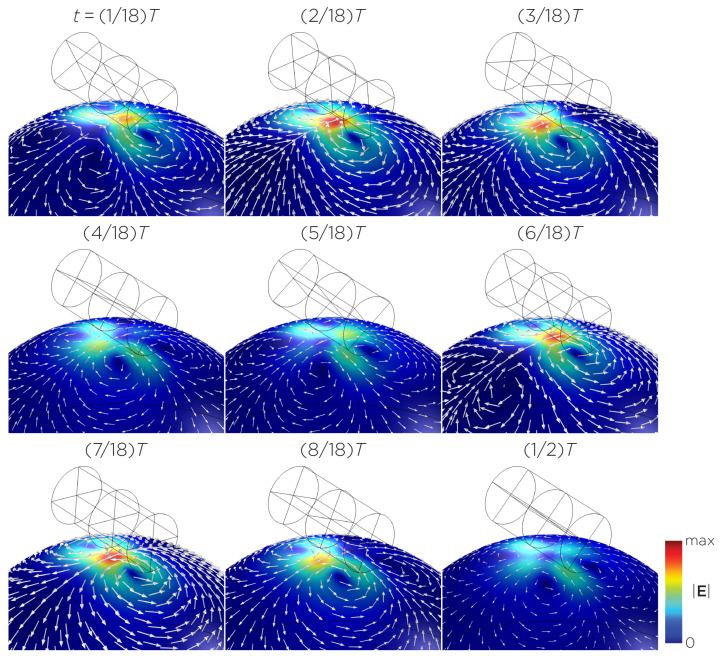
Half revolution of configuration H in steady state. The cylinder represents the magnet.

Figure 5 displays a bipolar E-field distribution in Model C. As the magnet rotates, the E-field distribution shifts from a four-leaf-clover pattern (when the magnetization direction is perpendicular to the spherical head at multiples of T/2) to a figure-8 pattern (at multiples of T/4). The peak induced E-field strength at the head’s surface measures approximately 0.13 V/m. Figure 6 (Model D) showcases another bipolar E-field distribution similar to Models A and B. Similarly, the circular pattern occurs when the magnetization directions are parallel to the spherical head. In this configuration, the peak induced E-field strength measures approximately 0.13 V/m. Additionally, Figure 7 (Model E) shows a bipolar E-field distribution with a similar pattern to Figure 5 (Model C), with a lower peak induced E-field strength of approximately 0.025 V/m. Figure 8 (Model F) demonstrates a quadrupolar E-field distribution. As the magnet rotates, the E-field distribution has the shape of a four-leaf clover that rotates. The peak induced E-field strength measures approximately 0.13 V/m. Figure 9 is another quadrupole E-field distribution, with similar E-field patterns to Models A, B, and D. In this configuration, the peak induced E-field strength is approximately 0.23 V/m. Figure 10 shows an eight-pole E-field distribution with a similar E-field distribution as Model C. The peak induced E-field strength measures approximately 0.14 V/m.

**Figure 11:**
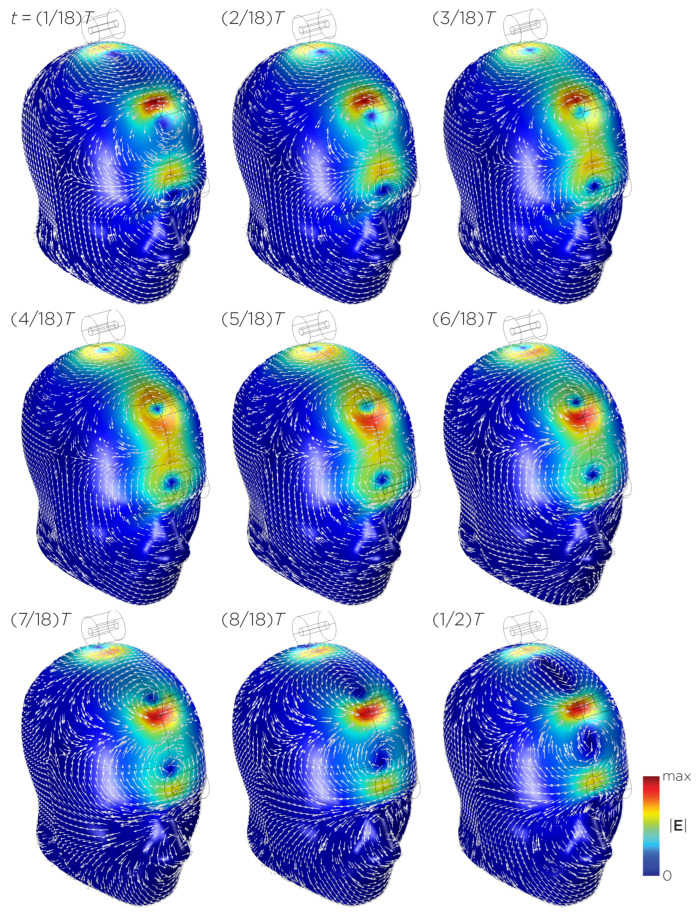
Half revolution of configuration I in steady state. The cylinders represent the magnets.

**Figure 12:**
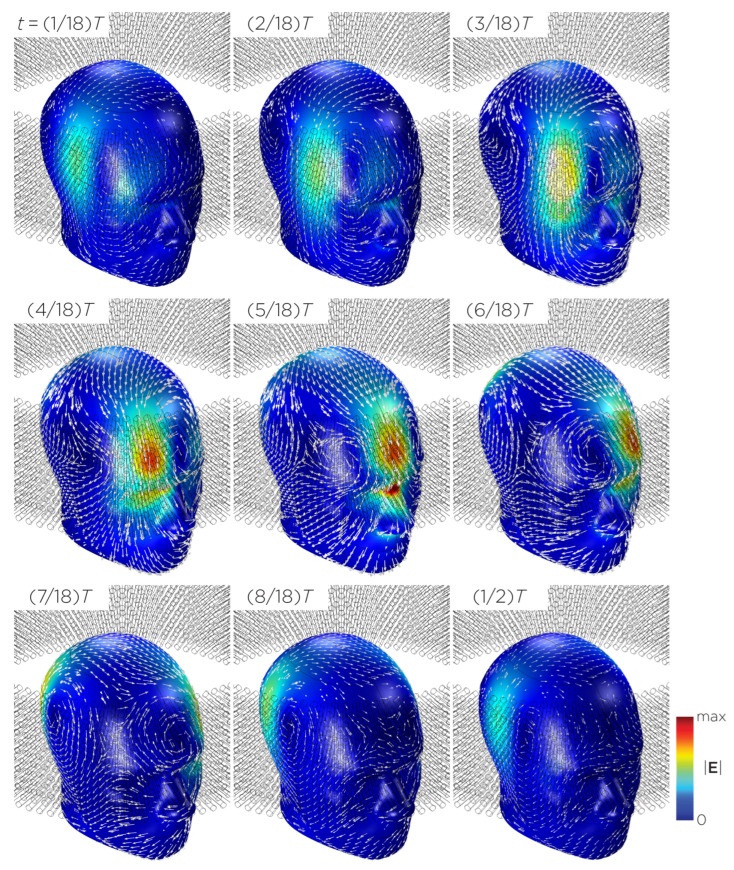
Half revolution of configuration J in steady state. The cylinders represent the magnets.

Figure 11 shows the E-field distribution of the full sTMS configuration in the SAM head model. The stimulation is broadly distributed over the midline frontal polar, medial frontal, and parietal regions. The peak induced E-field strength at the surface of the head is approximately 0.11 V/m. At a depth of 1.5 cm from the head surface, corresponding to the depth of the cortex, the E-field strength attenuates by approximately half. Figure 12 shows the E-field distribution of the wide-bore, low-frequency magnetic spinner. The stimulation is broadly distributed vertically of the head and rotates around the head as the device spins. The peak induced E-field strength at the surface of the head is approximately 0.0092 V/m.

### 3.2. Experimental Measurements

When comparing the E-field measurements to the computational results for Models A and B, similar values are reported in Table 2. Specifically, the maximum E-field strength for Models A and B was found to be approximately 0.39 V/m and 0.082 V/m when the magnets were spun at 349.9 rps and 10.1 rps, respectively. Model B, representing one single magnet in the sTMS system, induces a maximum E-field strength (0.082 V/m) which is lower than the maximum induced E-field strength of the sTMS system (0.11 V/m). The maximum induced E-field for the MagVenture TMS coil was measured to be approximately 401.5 V/m at a pulse frequency of 3448 Hz, corresponding to a pulse width of 290 μs. The measured induced E-field has a similar order of magnitude to previous simulations (370 V/m) [29].

## 4. Discussion

The distribution of the E-field induced by a single rotating magnet is influenced by several factors, including the dimensions and placement of the magnet(s), the number of poles, the direction of the magnetization, and the axis of rotation. Furthermore, the E-field strength is dependent on the rotational frequency and the surface field strength of the magnets. Our simulations revealed that the induced E-field strength on the head surface ranged from 0.0092 V/m to 0.52 V/m. The spatial pattern of the E-field varied between circular, figure-8, or four-leaf-clover shapes, depending on the relative orientation of the magnetization vector to the head model. For instance, in Model A, the E-field exhibits a circular pattern when the magnetization vector is parallel to the head and morphs into a figure-8 pattern when the magnetization vector is rotated perpendicular to the head. With more than one magnet, the E-fields from each magnet are summated according to the principle of superposition, resulting in more complex patterns and strengths depending on the arrangement and characteristics of the magnets (e.g., the distance between the magnets and their initial polarizations). Similarly, single-magnet configurations with multiple poles illustrated complex patterns and strengths, depending on the summated magnetization directions. In general, configurations with a figure-8 or a more localized E-field distribution resulted in a higher peak surface E-field strength, while those with a circular or more spread-out pattern induced lower peak surface E-field strength. This phenomenon mirrors the depth–focality trade-off observed in TMS coils, where the E-field strength in a more focal distribution decays more rapidly with distance compared to a more spread-out E-field distribution [30].

In the full sTMS model (Model I), the magnets were set to rotate at a fixed frequency of 10 rps. Since the induced E-field strength is linearly proportional to the rotational frequency, the field strengths at other frequencies can be easily calculated (Figure 3B). In practice, the sTMS system synchronizes the rotational frequency of the magnets to the IAF measured from EEG, which typically ranges between 8 and 13 Hz [9]. Jin and Phillips estimated the intensity of sTMS to be less than 1% that of conventional TMS [9]. This estimate was based on the ratio of the maximum rate of change in the magnetic field over time, dB/dt, between the sinusoidal waveform of sTMS and pulsed waveform of conventional TMS. However, this comparison did not account for the magnetic fields’ spatial characteristics and the head’s boundary conditions, which are crucial factors that affect the distribution and intensity of the induced E-field. Our simulation and measurement of the single magnet (Model B), as well as simulation of the full three-magnet array (Model I), yielded a peak E-field strength of approximately 0.11 V/m. This strength represents only 0.025% of conventional TMS at the surface of the head.

Synchronizing the exogenous subthreshold sinusoidal stimulation to the intrinsic alpha EEG rhythm was thought to be an important feature that underlies the mechanism of sTMS treatment for depression [6]. In the sTMS depression study, some participants did not receive stimulation at the correct IAF, which led to inferior outcomes compared to those treated at the correct IAF [7]. Secondary analysis showed that participants with a lower IAF exhibited the least clinical improvement [8]. It is important to note that since the induced E-field strength is directly proportional to the rotational frequency of the magnets, customizing the rotational frequency could introduce variability in the induced E-field strength across individuals. Consequently, a lower IAF would determine a lower rotational frequency of the magnets, leading to decreased E-field strength, thereby potentially confounding the interpretation of this finding. One potential solution to mitigate this confound is to employ electromagnets, such as those used in n-phase motors. In a three-phase motor, for example, three coil windings in the motor stator receive power from three alternating currents that are out of phase with each other by one-third of their cycle, creating a magnetic field that rotates, similar to that in a mechanically rotating permanent magnet system. The advantage of using electromagnets is that they independently control frequency and amplitude, as opposed to the fixed coupling of these variables in mechanical rotating systems.

Compared to the sTMS system, the TRPMS system produces an E-field strength which is approximately five times higher, achieved through the use of a stronger magnet and a higher rotational frequency. Both the sTMS and TRPMS systems fall within similar order of magnitude, which are significantly lower compared to conventional TMS. Inductive measurements by Helekar and colleagues estimated the maximum TRPMS stimulation intensity to be approximately 7% of the maximum conventional TMS output at a distance of 6.2 mm from the magnet or TMS coil [14]. First, these estimates are based on measurements made in the air and do not account for head boundary conditions, potentially overestimating the E-field strength. Second, the TMS waveform reported in Helekar et al. [14] does not resemble the conventional biphasic cosine waveform generated by the Magstim Rapid^2^ stimulator. This is possibly due to a lower sampling rate in their measurements, causing a distortion in the waveform, thus underestimating the peak value. Third, since smaller magnets have faster field attenuation with distance compared to larger magnets [30], the E-field strength of the TRPMS system at the depth of the cortex would be further overestimated. Our simulation and measurement for a single magnet in the TRPMS system showed that the peak E-field strength is approximately 0.1% of conventional TMS.

In the simulations of multipole magnets rotating at 10 rps (Model C–H), the E-field strengths are similar to that of the sTMS system, except for Model E, which exhibits an E-field strength of 0.025 V/m, which was approximately an order of magnitude lower. The E-field distribution from Watterson’s configurations demonstrated characteristics of multiple magnets. For example, Model C, representing the bipole configuration used in Watterson’s nerve stimulation experiments, exhibits a four-clover and a figure-8-shaped field pattern. The four-clover pattern emerges when the magnetization direction is perpendicular and shifts to a figure-8 pattern when the magnetization direction is parallel to the head model. Using this bipole configuration, Watterson and colleagues demonstrated the ability to achieve nerve activation at a rotational frequency of 230 rps [20]. According to their measurements, this resulted in an E-field strength of approximately 1 V/m, equivalent to 0.4% of the conventional TMS maximum output. In our finite element models, we use a rotational frequency of 10 rps to simulate the effect of multipolar magnet stimulation for brain stimulation. The 10 Hz frequency matches that of the sTMS model. Our simulation shows that this configuration induces an E-field strength of 0.13 V/m, approximately 0.032% of conventional TMS at the surface of the head. The multipolar magnets could be as effective as sTMS when used as part of a brain stimulation device.

In comparison to other proposed rotating magnetic systems, the wide-bore, low-frequency magnetic spinner (Model J), designed to induce alternating electric currents in biological tissues, induced the lowest and most nonfocal E-fields. This device generates a maximum magnetic field of 2.5 mT, resulting in a maximum induced E-field of 0.0092 V/m in the head model. With the installation of a magnetic yoke, which concentrates the magnetic flux to the inside of the bore, the measured magnetic field reaches 32 mT, bringing the induced E-field strength close to that of other devices. In terms of the spatial distribution, there are two E-field peaks located where the column of magnets reverses magnetization, e.g., where the magnetic field gradient is the highest. Since the induced field is more diffused, the field penetration is deeper compared to other smaller rotating magnetic configurations.

One potential advantage of utilizing rotating permanent magnets is the ability to create portable, cost-effective devices compared to conventional TMS [20]. Depending on the magnet strength and rotational frequency, the E-field strengths in the sTMS, TRPMS, and Watterson multipolar systems are comparable to other forms of low field stimulation, including low field magnetic stimulation (LFMS) [31], transcranial current stimulation (tCS) [32,33], and low-intensity repetitive magnetic stimulation (LI-rMS) [34,35]. Low field stimulation has been shown to induce changes at the cellular and molecular levels. For example, in an in vitro model, LI-rMS has been shown to alter cellular activation and gene expression in an organotypic hindbrain explant and in a stimulation frequency-specific manner [34]. Dufor and colleagues reported the induced E-field strengths of this device to be between 0.05 and 0.075 V/m [36]. Similarly, LI-rMS delivered during visually evoked activity increased the densities of parvalbumin-expressing GABAergic interneurons in an adult mouse visual cortex [37]. These findings suggest that the low field strengths produced by rotating permanent magnets might be biologically active, warranting further investigation to evaluate their potential therapeutic value.

Achieving higher field strengths through increased rotational speeds of the magnets is feasible. However, it is important to consider the low-pass filtering property of the neuronal membrane, rapid changes in voltage are not transmitted as efficiently across the membrane, diminishing the effect of high frequency stimulation [38]. Additionally, the interaction between field strength and excitation frequency could be nonlinear, as demonstrated in a transcranial alternating current stimulation (tACS) study [39,40]. For example, when 140 rps tACS is applied to the motor cortex, a low current amplitude of 0.4 mA results in a reduction in motor evoked potential (MEP) amplitudes; intermediate amplitudes of 0.6 mA and 0.8 mA showed no effect on MEP, and a high amplitude of 1 mA resulted in the enhancement of MEP amplitudes [39].

Several limitations should be acknowledged when interpreting this work. First, the simulations were not performed on realistic head models. Our models assume a simplified geometry with homogeneous, isotropic conductivity to better illustrate the spatial field distribution. Realistic head models consist of several tissue types with varying conductivities, the values of which are frequency dependent [22,23]. However, at the low frequency range that we are operating in (<1 kHz), tissue conductivity values remain relatively stable [22,23]. In addition, cortical folding in realistic head models can increase the maximum E-field strength compared to spherical head models [32,41]. It has been shown, for example, that skin conductivities’ variation can result in minor changes in E-field strength induced by TMS [42]. Future work could consider integrating realistic head models to better represent accurate head anatomy and the variations in E-field strengths across individuals. The second limitation of our work is that we did not perform a high-resolution spatial sampling of the E-field in the phantom to characterize the full spatial distribution, which varies over time. Our focus was simply on measuring the peak E-field strength to validate the simulation magnitudes and compare them to previously conducted measurements and to conventional TMS.

## 5. Conclusions

In this study, we evaluated the E-field characteristics of the sTMS system, TRPMS system, and other configurations of rotating magnets using finite element modeling and phantom head measurements. Our findings indicate that the maximum induced E-field strength on the head surface ranged from 0.0092 V/m to 0.52 V/m, which is on the order of 0.1% of the field strength induced by conventional TMS. Furthermore, we demonstrate that E-field strength depends on rotational frequency, which represents a previously unappreciated confound in clinical trials that seek to synchronize rotational frequency to individual endogenous oscillatory activity. Future research directions include conducting simulations of rotating magnetic stimulation on anatomically accurate head models, which would be based on individual brain imaging data, as well as optimizing treatment parameters such as stimulation frequency and magnet placement. Additionally, it is essential to gather direct electrophysiological data to corroborate the hypothesized mechanism of action of these stimulation systems.

## Figures and Tables

**Table 1:** Magnet specifications (magnet dimensions, magnetization directions, magnetic flux densities, rotational frequency) and the maximum induced B- and E-field strength for Models A–J.

Model	Dimensions (cm)	Magnetization Direction	*B*_r_ (T)	Rotational Frequency (rps)	Maximum |B| (mT)	Maximum |E| (V/m)
A	Cylinder×1 od=0.635 h=0.9525	Axial	1.48	350	94.1	0.52
B	Ring×1 od=2.54 id=0.635 h=2.54	Diametrical	1.32	10	334.8	0.098
C	Cylinder×1 2 segments od=3, h=6	Diametrical, multipole	1.48	10	462.8	0.13
D	Cylinder×1 2 segments od=3, h=3	Diametrical, multipole	1.48	10	209.8	0.13
E	Cylinder×1 2 segments od=3, h=3	Axial, multipole	1.48	10	134.1	0.025
F	Cylinder×1 4 segments od=5, h=1	Axial, multipole	1.48	10	2	0.13
G	Cylinder×1 4 segments od=3, h=3	Radial, multipole	1.48	10	353.7	0.23
H	Cylinder×1 8 segments od=3, h=6	Radial, multipole	1.48	10	350.6	0.14
I	Ring×3 od=2.54 id=0.635 h=2.54	Diametrical	1.32	10	354.7	0.11
J	Cylinder×1224 od=0.3175 h=2.54 Arrayid=30 12 layers s=1.905	Axial	1.48	13.3	2.5	0.0092

od: outer diameter; id: inner diameter; h: height; s: layer separation.

**Table 2:** E-field measurements compared to the computational measurements.

Configuration	Measured Rotational/Pulse Frequency (rps, Hz)	Measured Maximum |E| (V/m)	Simulated Maximum |E| (V/m)
Model A	349.9	0.39	0.52
Model B	10.1	0.082	0.098
TMS	3448	401.5	370 [29]

## Data Availability

The raw data supporting the conclusions of this article will be made available by the authors on request.

## References

[1] Cohen S.L., Bikson M., Badran B.W., George M.S. (2022). A visual and narrative timeline of US FDA milestones for Transcranial Magnetic Stimulation (TMS) devices. Brain Stimul..

[2] Rizvi S., Khan A.M. (2019). Use of transcranial magnetic stimulation for depression. Cureus.

[3] Phillips J.W., Jin Y. (2015). Systems and Methods for Neuro-EEG Synchronization Therapy. U.S. Patent.

[4] Watterson P.A. (2014). Device Including Moving Magnet Configurations. U.S. Patent.

[5] Helekar S.A., Voss H.U. (2019). Method and apparatus for providing trancranial magnetic stimulation (TMS) to a individual. U.S. Patent.

[6] Leuchter A.F., Cook I.A., Jin Y., Phillips B. (2013). The relationship between brain oscillatory activity and therapeutic effectiveness of transcranial magnetic stimulation in the treatment of major depressive disorder. Front. Hum. Neurosci..

[7] Leuchter A.F., Cook I.A., Feifel D., Goethe J.W., Husain M., Carpenter L.L., Thase M.E., Krystal A.D., Philip N.S., Bhati M.T. (2015). Efficacy and safety of low-field synchronized transcranial magnetic stimulation (sTMS) for treatment of major depression. Brain Stimul..

[8] Philip N.S., Leuchter A.F., Cook I.A., Massaro J., Goethe J.W., Carpenter L.L. (2019). Predictors of response to synchronized transcranial magnetic stimulation for major depressive disorder. Depress. Anxiety.

[9] Jin Y., Phillips B. (2014). A pilot study of the use of EEG-based synchronized transcranial magnetic stimulation (sTMS) for treatment of major depression. BMC Psychiatry.

[10] Cook I.A., Wilson A.C., Corlier J., Leuchter A.F. (2019). Brain activity and clinical outcomes in adults with depression treated with synchronized transcranial magnetic stimulation: An exploratory study. Neuromodulation.

[11] Phillips J.W., Jin Y. (2017). Devices and Methods of Low Frequency Magnetic Stimulation Therapy. U.S. Patent.

[12] Philip N.S., Aiken E.E., Kelley M.E., Burch W., Waterman L., Holtzheimer P.E. (2019). Synchronized transcranial magnetic stimulation for posttraumatic stress disorder and comorbid major depression. Brain Stimul..

[13] Helekar S.A., Voss H.U. (2016). Transcranial brain stimulation with rapidly spinning high-field permanent magnets. IEEE Access.

[14] Helekar S.A., Convento S., Nguyen L., John B.S., Patel A., Yau J.M., Voss H.U. (2018). The strength and spread of the electric field induced by transcranial rotating permanent magnet stimulation in comparison with conventional transcranial magnetic stimulation. J. Neurosci. Methods.

[15] Khavari R., Tran K., Helekar S.A., Shi Z., Karmonik C., Rajab H., John B., Jalali A., Boone T. (2022). Noninvasive, individualized cortical modulation using transcranial rotating permanent magnet stimulator for voiding dysfunction in women with multiple sclerosis: A pilot trial. J. Urol..

[16] Greene E., Thonhoff J., John B.S., Rosenfield D.B., Helekar S.A. (2021). Multifocal noninvasive magnetic stimulation of the primary motor cortex in yype 1 myotonic dystrophy - a proof of concept pilot study. J. Neuromuscul. Dis..

[17] Jang Y., Tran K., Shi Z., Christof K., Choksi D., Salazar B.H., Lincoln J.A., Khavari R. (2022). Predictors for outcomes of noninvasive, individualized transcranial magnetic neuromodulation in multiple sclerosis women with neurogenic voiding dysfunction. Continence.

[18] Tran K., Shi Z., Karmonik C., John B., Rajab H., Helekar S.A., Boone T., Khavari R. (2021). Therapeutic effects of non-invasive, individualized, transcranial neuromodulation treatment for voiding dysfunction in multiple sclerosis patients: Study protocol for a pilot clinical trial. Pilot Feasibility Stud..

[19] Wide-Bore Low-Frequency Magnetic Spinner for Non-Contact E-Field Generation in a Tissue via Faraday’s Law of Induction. https://www.nevaelectromagnetics.com/wide-bore-low-freq-generator-type1.

[20] Watterson P.A., Nicholson G.M. (2016). Nerve-muscle activation by rotating permanent magnet configurations. J. Physiol..

[21] Heller L., van Hulsteyn D.B. (1992). Brain stimulation using electromagnetic sources: Theoretical aspects. Biophys. J..

[22] Gabriel S., Lau R., Gabriel C. (1996). The dielectric properties of biological tissues: II. Measurements in the frequency range 10 Hz to 20 GHz. Phys. Med. Biol..

[23] Gabriel S., Lau R., Gabriel C. (1996). The dielectric properties of biological tissues: III. Parametric models for the dielectric spectrum of tissues. Phys. Med. Biol..

[24] Temperature and Neodymium Magnets. https://www.kjmagnetics.com/blog.asp?p=temperature-and-neodymium-magnets.

[25] Rotating Machinery 3D Tutorial. https://doc.comsol.com/6.0/doc/com.comsol.help.models.acdc.rotating_machinery_3d_tutorial/models.acdc.rotating_machinery_3d_tutorial.pdf.

[26] Glover P.M., Bowtell R. (2007). Measurement of electric fields due to time-varying magnetic field gradients using dipole probes. Phys. Med. Biol..

[27] Ramon C., Garguilo P., Fridgeirsson E.A., Haueisen J. (2014). Changes in scalp potentials and spatial smoothing effects of inclusion of dura layer in human head models for EEG simulations. Front. Neuroeng..

[28] Sauerheber R., Heinz B. (2015). Temperature effects on conductivity of seawater and physiologic saline, mechanism and significance. Chem. Sci. J..

[29] Smith J.E., Peterchev A.V. (2018). Electric field measurement of two commercial active/sham coils for transcranial magnetic stimulation. J. Neural. Eng..

[30] Deng Z.D., Lisanby S.H., Peterchev A.V. (2013). Electric field depth–focality tradeoff in transcranial magnetic stimulation: Simulation comparison of 50 coil designs. Brain Stimul..

[31] Rohan M.L., Yamamoto R.T., Ravichandran C.T., Cayetano K.R., Morales O.G., Olson D.P., Vitaliano G., Paul S.M., Cohen B.M. (2014). Rapid mood-elevating effects to low field magnetic stimulation in depression. Biol. Psychiatry.

[32] Miranda P.C., Mekonnen A., Salvador R., Ruffini G. (2013). The electric field in the cortex during transcranial current stimulation. Neuroimage.

[33] Guidetti M., Arlotti M., Bocci T., Bianchi A.M., Parazzini M., Ferrucci R., Priori A. (2022). Electric fields induced in the brain by transcranial electric stimulation: A review of in vivo recordings. Biomedicines.

[34] Grehl S., Martina D., Goyenvalle C., Deng Z.D., Rodger J., Sherrard R.M. (2016). In vitro magnetic stimulation: A simple stimulation device to deliver defined low intensity electromagnetic fields. Front. Neural. Circuits.

[35] Moretti J., Terstege D.J., Poh E.Z., Epp J.R., Rodger J. (2022). Low intensity repetitive transcranial magnetic stimulation modulates brain-wide functional connectivity to promote anti-correlated c-Fos expression. Sci. Rep..

[36] Dufor T., Grehl S., Tang A.D., Doulazmi M., Traoré M., Debray N., Dubacq C., Deng Z.D., Mariani J., Lohof A.M. (2019). Neural circuit repair by low-intensity magnetic stimulation requires cellular magnetoreceptors and specific stimulation patterns. Sci. Adv..

[37] Makowiecki K., Garrett A., Harvey A.R., Rodger J. (2018). Low-intensity repetitive transcranial magnetic stimulation requires concurrent visual system activity to modulate visual evoked potentials in adult mice. Sci. Rep..

[38] Brunel N., van Rossum M.C.W. (2007). Quantitative investigations of electrical nerve excitation treated as polarization. Biol. Cybern..

[39] Moliadze V., Atalay D., Antal A., Paulus W. (2012). Close to threshold transcranial electrical stimulation preferentially activates inhibitory networks before switching to excitation with higher intensities. Brain Stimul..

[40] Numssen O., Zier A.L., Thielscher A., Hartwigsen G., Knösche T.R., Weise K. (2021). Efficient high-resolution TMS mapping of the human motor cortex by nonlinear regression. Neuroimage.

[41] Thielscher A., Opitz A., Windhoff M. (2011). Impact of the gyral geometry on the electric field induced by transcranial magnetic stimulation. Neuroimage.

[42] Colella M., Paffi A., De Santis V., Apollonio F., Liberti M. (2021). Effect of skin conductivity on the electric field induced by transcranial stimulation techniques in different head models. Phys. Med. Biol..

